# Fucosylation in Urological Cancers

**DOI:** 10.3390/ijms222413333

**Published:** 2021-12-11

**Authors:** Kazutoshi Fujita, Koji Hatano, Mamoru Hashimoto, Eisuke Tomiyama, Eiji Miyoshi, Norio Nonomura, Hirotsugu Uemura

**Affiliations:** 1 Department of Urology, Kindai University Faculty of Medicine, 377-2, Ohno-Higashi, Sayama 589-8511, Osaka, Japan; hashimot@med.kindai.ac.jp (M.H.); huemura@med.kindai.ac.jp (H.U.); 2 Department of Urology, Osaka University Graduate School of Medicine, Suita 565-0871, Osaka, Japan; hatano@uro.med.osaka-u.ac.jp (K.H.); tomiyama@uro.med.osaka-u.ac.jp (E.T.); nono@uro.med.osaka-u.ac.jp (N.N.); 3 Department of Molecular Biochemistry and Clinical Investigation, Osaka University Graduate School of Medicine, Suita 565-0871, Osaka, Japan; emiyoshi@sahs.med.osaka-u.ac.jp

**Keywords:** fucosylation, fucosyltransferase, prostate cancer, bladder cancer, kidney cancer

## Abstract

Fucosylation is an oligosaccharide modification that plays an important role in immune response and malignancy, and specific fucosyltransferases (FUTs) catalyze the three types of fucosylations: core-type, Lewis type, and H type. FUTs regulate cancer proliferation, invasiveness, and resistance to chemotherapy by modifying the glycosylation of signaling receptors. Oligosaccharides on PD-1/PD-L1 proteins are specifically fucosylated, leading to functional modifications. Expression of FUTs is upregulated in renal cell carcinoma, bladder cancer, and prostate cancer. Aberrant fucosylation in prostate-specific antigen (PSA) could be used as a novel biomarker for prostate cancer. Furthermore, elucidation of the biological function of fucosylation could result in the development of novel therapeutic targets. Further studies are needed in the field of fucosylation glycobiology in urological malignancies.

## 1. Introduction

Fucosylation is an oligosaccharide modification that plays an important role in immune response and malignancies. Changes in glycosylation status affect cellular function via glycosylated proteins, such as growth factor receptors and adhesion molecules.

To date, advances in glycomics have identified several types of biomarkers, including fucosylation-related factors, that are associated with certain types of cancers.

One of the representative fucosylation-related cancer biomarkers is fucosylated alpha-fetoprotein (AFP), which is named the AFP-L3 fraction. It has been widely used as a specific cancer biomarker for hepatocellular carcinoma (HCC) since 1996 in Japan and 2005 in the United States [1,2] because it is more specific than alpha-fetoprotein. Another famous one is sialic Lewis a (sLe^a^), also known as CA19-9, which has been clinically used as a cancer biomarker for pancreatic cancer [3].

**Figure 1:**
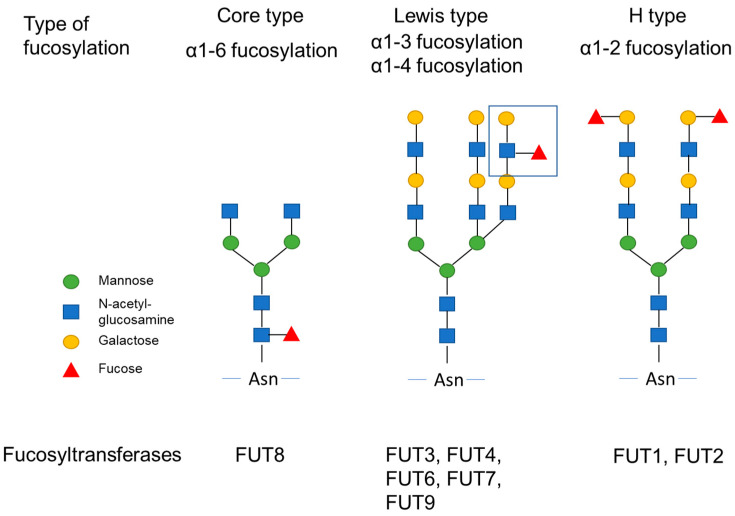
Fucosylation and fucosyltransferases. Three types of fucosylation, α1-2 fucose (H type), α1-3/α1-4 fucose (Lewis type), and α1-6 fucose (core type) are shown. Each fucosylation are catalyzed by specific fucosyltransferases.

Glycosylation, including fucosylation of the protein, occurs within the Golgi endoplasmic reticulum. Fucosylation is dependent on fucosyltransferase (FUT) activity and the level of its donor substrate, guanosine diphosphate (GDP)-fucose synthetic enzyme. In addition, GDP-mannose-4,6-dehydratase (GMDS) is a key enzyme involved in the synthesis of GDP-fucose [4]. Three types of fucosylation exist: α1-2 fucose (H type), α1-3/α1-4 fucose (Lewis type), and α1-6 fucose (core type) (Figure 1). Each type of fucosylation is catalyzed by specific FUTs [5,6,7,8]. Eleven different FUTs are classified into four groups. FUT1 and FUT2 catalyze the synthesis of α1-2 fucose, FUT3, 4, 5, 6, 7, and 9 catalyze the synthesis of α1-3/α1-4 fucose, and only FUT8 catalyzes the synthesis of α1-6 fucose [5]. FUT activity has not been reported for either FUT10 or FUT11.

This review will discuss the importance of fucosylation as a biomarker in malignancies, especially urological cancers, and the possible biological and clinical role of fucosylation based on recent findings.

## 2. Biological Function of Fucosyltransferases and GDP-Fucose Synthetic Enzyme

Fucosylation is the enzymatic attachment of L-Fucose to oligosaccharides on glycoproteins and glycolipids or proteins. It is one of the most important post-translational modifications, which can confer unique functions to oligosaccharides or proteins. L-fucose is equivalent to 6-deoxy-L-galactose and has two structural differences from other hexoses in mammals. One is the lack of a hydroxyl group on the carbon at the 6-position and the other is the L-configuration. Fucosylation is closely associated with various physiological events and is reported to be upregulated in several types of cancers. Fucosylation is regulated by FUTs, and FUTs regulate cancer proliferation, invasiveness, and resistance to chemotherapy by modifying the glycosylation of signaling receptors. Each FUT has been reported to play a significant role in various types of cancers (Table 1).

FUT1 and FUT2 encode α1-2 FUTs. Glycan products of FUT1 and FUT2, such as Globo H and Lewis Y, are highly expressed in malignant tissues. Overexpression of FUT1 or FUT2 promotes the migration and invasion of tumor cells in breast cancer cell lines and metastasis in vivo, whereas FUT1 or FUT2 knockdown reduces Globo H and decreases mesenchymal-like markers, such as fibronectin, vimentin, and twist [9]. FUT1 expression is associated with chemotherapy resistance in ovarian cancer through interaction with apoptotic pathways [10,11].

FUT3 overexpression promotes the fucosylation of transforming growth factor (TGF)βR-I, resulting in the activation of the TGF-β signaling pathway in colorectal cancer [10]. The FUT3 gene is also known as the Lewis gene, and the high expression of FUT3 and its product, tetrasaccharide Sialic Lewis x (sLex), has been reported in several types of malignant tumors, such as breast cancer [12], pancreatic cancer [13], ovarian carcinoma, and colorectal cancer [14]. sLe^x^ is the well-known ligand of E-selectin [15], which mediates cell–cell adhesion. This adhesion is necessary for leukocyte rolling on the endothelium when inflammation takes place, but tumor cells utilize its mechanism to extravasate to distant places during metastasis, resulting in the metastasis of cancer [16]. A recent study reported that the abilities of proliferation, invasion, and migration in gastric carcinoma can be inhibited by FUT3 gene silencing [17]. In addition, FUT3 also specifically catalyzes another well-known fucosylated glycan, sialic Lewis a (sLea, CA19-9). sLe^a^ is another ligand for E-selectin and a serum biomarker of pancreatic cancer and colorectal cancer in the prediction of prognosis and surveillance of recurrence [18].

FUT4 is frequently upregulated in lung cancer, and FUT4 knockdown increases chemosensitivity to cisplatin by suppressing FOXO1-induced apoptosis [19]. FUT4 overexpression promotes lung cancer invasion, migration, epithelial-mesenchymal transition (EMT), and cell adhesion. Genome-wide RNA-seq and immunoprecipitation–mass spectrometry showed that FUT4 is associated with membrane trafficking, cell cycle, and major oncogenic signaling pathways [20]. Metastasis-associated lung adenocarcinoma transcript 1(MALAT1) in exosomes increased FUT4-mediated fucosylation and phosphorylation of the PI3K/Akt/mTOR pathway and promoted invasion and metastasis in colon cancer cells [21].

FUT5 or FUT6 expression modulates the activity of the PI3K/Akt signaling pathway in colorectal cancer cells [22]. FUT7 is overexpressed in follicular thyroid carcinoma and is associated with a poor prognosis. The extent of glycan α1-3 fucosylation on epidermal growth factor receptor (EGFR) was positively correlated with EGFR activation. FUT7 enhances EGF-induced progression of follicular thyroid carcinoma cells through mitogen-activated protein kinase (MAPK) and phosphatidylinositol-3-kinase (PI3K)/Akt signaling pathways [23]. In bladder cancer, FUT7 expression is correlated with tumor-infiltrating lymphocytes and promotes tumor proliferation, migration, invasion, and EMT [24].

FUT8 is the only FUT that catalyzes core fucosylation (α1-6 fucosylation) [25]. Core fucosylation of TGF-β receptor by FUT8 promotes TGF-β signaling and epithelial-mesenchymal transition in breast cancer cells [26]. In mice, FUT8 knockout induces severe growth retardation, early death during postnatal development, and emphysema-like changes in the lungs. Core fucosylation plays an important role in growth factor receptors, such as TGF-β1 and EGFR-mediated biological functions [27]. Blocking core fucosylation of TGF-βRII and ALK5 in renal tubular cells inhibits TGF-β1 signaling by suppressing the phosphorylation and translocation of Smad2/3 to the nucleus, resulting in the inhibition of EMT [28]. FUT8 overexpression is also observed in cancer-associated fibroblasts (CAFs) in non-small-cell lung carcinoma, and FUT8 in CAFs promotes the formation of an invasive and malignant tumor microenvironment via core fucosylation of EGFR [29]. Overexpression of FUT8 in prostate cancer cells reduces the number of extracellular vesicles secreted by prostate cancer cells and increases the abundance of proteins associated with cell motility and prostate cancer metastasis [30].

In colon cancer cells, FUT9 expression is associated with the expression of genes related to cancer stemness. Lewis X, the markers of stemness (CD44 expression, ALDH, Sox2), and tumor-sphere formation, chemoresistance to 5-FU treatment, and proliferation of tumor in vivo were increased in FUT9-expressing murine colon adenocarcinoma cells compared with control cells [31].

FUT activity has not been confirmed for either FUT10 or FUT11 [2], but a meta-analysis of microarray data showed that FUT11 expression was associated with tumor progression in clear cell renal cell carcinoma [32].

### GDP-Fucose Synthetic Enzyme

GDP-fucose synthetic enzyme, also known as TSTA3, is the key enzyme involved in fucosylation. TSTA3 produces GDP-L-fucose, which is the only donor of fucosylation. TSTA3 is amplified in esophageal squamous cell carcinoma, and TSTA3 overexpression results in increased cell invasion and tumor metastasis. TSTA3 may play a role in promoting invasion by increasing the core fucosylation of LAMP2 and the terminal fucosylation of ERBB2 [33]. In glioblastoma cell lines, Golgi phospholipoprotein 3 (GOLPH3) regulates EGFR via terminal sialylation and fucosylation. Changes in the glycosylation pattern of EGFR lead to changes in its localization and tracking [34]. In a breast cancer cell line, the fucosylation inhibitor 2-fluorofucoseS decreases NF-κB activity by increasing IκBα [35].

## 3. Fucosylation in Cancer Immunology

The development of immune checkpoint inhibitors has changed the treatment strategy for advanced renal cell carcinoma and urothelial carcinoma [36]. Recently, the evaluation of glycosylation profiles has gained increasing interest in several types of cancers, especially in immunotherapeutic targets, such as PD-1 and PD-L1 [37,38,39]. The *N*-glycans of PD-1 and PD-L1 are highly core fucosylated, and fucosylation also plays an important role in the regulation of PD-1 and PD-L1 functions. Inhibition of FUT8 by genetic ablation or inhibitors reduces cell-surface expression of PD-1 and enhances T cell activation, resulting in the improvement of anti-tumor response in mice [40].

The T cell receptor is also a highly core fucosylated glycoprotein. The core fucosylation of CD4^+^ T cells is significantly increased in systemic lupus erythematosus patients, and loss of FUT8 reduced CD4+ T cell activation [41]. Levels of core fucosylation of T cell receptors are increased in patients with inflammatory bowel disease, and core fucosylation of the T cell receptor is required for T cell signaling, the production of inflammatory cytokines, and induction of colitis in mice [42]. Core fucosylation is required for signal transduction via T cell receptors (TCRs), and the ablation of core fucosylation results in abnormal T cell development in the thymus due to attenuated signaling via TCRs [43]. The formation of lung adenocarcinoma is markedly reduced in FUT8^−/−^OT-I mice. Removal of core fucosylation from PD-1 impaired its expression on CD8+ cytotoxic T lymphocytes (CTLs), resulting in enhanced CTL activation and cytotoxicity, leading to tumor eradication. Loss of core fucosylation significantly enhanced PD-1 ubiquitination, leading to the degradation of PD-1 in the proteasome [44]. Inflammatory cytokine production by macrophages was suppressed in FUT8-deficient mice. Core fucose is essential for CD14-dependent toll-like receptor (TLR) 4 and TLR2 signaling in mouse macrophages [45]. Levels of α1-3 fucosylated AGP (fAGP) increased along with disease progression and decreased in response to chemotherapy treatments in patients with lung cancer. Thus, Serum fAGP level is a biomarker for predicting the clinical efficacy of immunotherapy with nivolumab [46].

In addition, it is reported that Immunoglobulin G (IgG) is also highly core fucosylated. Loss of fucosylation also has significant biological consequences toward IgG antibodies. IgG antibodies are crucial for protection against invading pathogens. A highly conserved N-linked glycan within the IgG-Fc tail is essential for IgG function and the lack of IgG core fucosylation results in 50–100 times higher activity of antibody-dependent cellular cytotoxicity [4]. Therefore, defucosylated IgG antibodies are already used in anticancer therapies for their increased activity through Fc receptors (FcγRIIIa) [47,48]. Another study also reported that fucosylation affects tumor immune surveillance and a deficiency of fucosylation leads to escape from NK cell-mediated tumor immune surveillance through regulating death-ligand-induced apoptosis via tumor-necrosis-factor-related apoptosis-inducing ligand (TRAIL) signaling [4].

## 4. Fucosylation in Renal Cell Carcinoma

Renal cell carcinoma (RCC) accounts for 3% of all malignancies in the United States. If detected in the early stage, RCC can be cured by surgery, with a 5-year survival rate of 70–90%. However, locally advanced disease has a great risk of recurrence, and metastatic disease is often fatal with a 5-year survival rate of less than 10%. RCC is usually detected incidentally by ultrasonography or computed tomography (CT), but there are no specific biomarkers for early detection and/or prediction of disease recurrence.

Metabolically, many transcriptomic and proteomic studies have reported the increased glucose and glutamine utilization associated with the Warburg effect in clear cell RCC (ccRCC), the most prevalent subtype of RCC [49,50]. The alternation of glucose and glutamine utilization may have effects on cell surface glycosylation including fucosylation in RCC tissue since the biosynthesis of the sugar components of glycoproteins, proteoglycans, and glycolipids are reportedly linked with glucose and glutamine metabolism.

As mentioned above, fucosylation is known to affect various cellular functions such as cell–cell adhesion, signal transduction, and immune recognition in malignant tumors. Vascular-growth-factor-targeted therapy and immunotherapy are the major therapeutic targets in RCC; changes in fucosylations are expected to have an impact on potential therapeutic effects.

Richard R. Drake et al. assessed the N-glycosylation patterns and compositional differences between tumor and non-tumor regions of ccRCC specimens using an imaging mass spectrometry approach and transcriptomic gene array data. Their results showed that multiantennary N-glycans with bisecting N-acetylglucosamine and multiple fucose residues, which are abundant in the proximal tubule, are not detected in ccRCC tissues, whereas multiple tumor-specific N-glycans with tri- and tetra-antennary structures and various levels of fucosylation and sialylation were present in ccRCC tissues. In addition, the analysis of transcriptomic gene array data revealed that the expression of the FUT3 and FUT6 genes coding fucosyltransferases responsible for α1-3/α1-4 fucosylation were significantly decreased in all ccRCC tissues compared with matched non-malignant tissues [51].

Tousi et al. focused on N-glycosylation of clusterin (apolipoprotein J), which is known to be upregulated in different types of RCC cell lines and tissues, and N-glycosylation of plasma clusterin in patients with RCC was examined using multi-dimensional high-performance liquid chromatography. The plasma levels of bi-antennary digalactosyl disialylated (A2G2S(3)2) glycans were significantly decreased, whereas the levels of a core fucosylated bi-antennary digalactosyl disialylated glycan (FA2G2S(6)2) and tri-antennary trigalactosyl disialylated glycan (A3G3S(6)2) were increased in the plasma samples after radical nephrectomy. Core fucosylation occurred in glycans of clusterin in the event of RCC [52]. The same group also assessed the individual N-glycosylation sites of plasma clusterin from patients with ccRCC using liquid chromatography followed by Tandem mass spectrometry. The core fucosylated biantennary digalactosylated disialylated (FA2G2S2) glycan in clusterin was reduced in the plasma of patients with clear cell RCC compared with plasma after nephrectomy [53]. Meng, L et al. performed immunohistochemical analysis of FUT3 expression in ccRCC and showed that high expression of FUT3 was associated with poor prognosis in ccRCC patients who underwent radical nephrectomy. FUT3 is an enzyme with α1-3 FUT and α1-4 FUT activities and catalyzes the Lewis blood group antigen [54].

The biological function of fucosylation in RCC remains to be elucidated, but these changes in fucosylation associated with RCC can be used to distinguish between cancer and non-cancer samples and suggest the potential for new therapeutic interventions and diagnostic markers

## 5. Fucosylation in Urothelial Carcinoma

Bladder cancer is the most frequently diagnosed urinary tract carcinoma, and urothelial carcinoma accounts for 90% of all bladder cancers. Despite significant advancements in medical treatment, bladder cancer detected at later stages has a poor prognosis [55]. There are also problems with non-muscle-invasive bladder cancer. The high recurrence rate after transurethral resection and progression to muscle-invasive disease is a challenging problem for patients [56]. Cystoscopy remains the gold standard tool for diagnosis and follow-up monitoring of the disease, but it is an invasive and costly procedure. As well as cystoscopy, urinary cytology has an established role in bladder cancer diagnosis and follow-up monitoring. However, it has poor sensitivity, particularly for low-grade tumors, and does not serve as a prognostic tool [57]. Although new biomarkers, such as Bladder EpiCheck, have been developed [58], there is an urgent need for the development and implementation in clinical practice of a potential therapeutic target and a non-invasive diagnostic biomarker for bladder cancer.

Core fucosylated *N*-glycans have been reported to be highly expressed, whereas terminal fucosylation of *N*-glycans is underexpressed in bladder cancer cell lines [59]. The abundant core fucosylated *N*-glycans in bladder cancer might be associated with prognosis or become a promising target molecule for future therapy. Terminal fucosylation occurs in *N*- and *O*-glycans through α1-2, α1-3, and α1-4 linkages, which are induced by FUTs ranging from FUT1 to FUT11, except for FUT8. Fucosylation by FUT1 through α1-2 linkages promotes bladder cancer progression [60]. However, the involvement of other FUTs in bladder cancer has not been reported, and further investigation is required. Lewis antigens found on cancer cell surfaces are another type of fucosylation product of oligosaccharides. Lewis X antigen (3-fucosyl-N-acetyl-lactosamine, CD15) is not detected in normal urothelial cells but is overexpressed in the malignant urothelium. It was reported that the diagnostic accuracy of bladder cancer detection can be enhanced by the Lewis X antigen [61]. Furthermore, the expression of Lewis X antigen in bladder cancer increases in proportion to stage, grade, and metastatic potential [62]. In contrast, lower expression of Lewis X antigen on human neutrophil glycoproteins is associated with better prognosis in patients with bladder cancer after total cystectomy [63].

Moreover, it is known that growth factor receptor signaling drives cancer progression. Other cancer types modulate growth factor receptor signaling, such as TGFβ, EGFR, vascular endothelial growth factor receptor, and c-Met, through fucosylation. Although bladder cancer progression is correlated with these signaling pathways [64,65], the relationship between fucosylation and bladder cancer progression has not been fully investigated. Jia Guo et al. compared N-glycan profiles of TGFβ-treated vs. control bladder cancer cells by MALDI-TOF/TOF-MS and revealed that fucosylation was increased in TGFβ-treated cells. This finding was consistent with the results of lectin microarray analysis, which showed that the Fucα1-2Galβ1-4GlcNAc structure recognized by Lectin ulex europaeus agglutinin-I (UEA-I) was approximately two-fold higher in TGFβ-treated cells than in control cells. These results may suggest that fucosylation plays an important role in TGFβ-induced EMT of bladder cancer [66].

Terminal fucosylation of oligosaccharides mediated by FUT1 is correlated with bladder cancer progression. It should be investigated whether abundant expression of core fucosylation mediated by FUT8 in bladder cancer patients could be a predictor of prognosis. Although growth factor receptor signaling activated by fucosylation has been reported in other cancer types, it has not been investigated in bladder cancer. Further research in this field is required.

As a biomarker for bladder cancer, fucosylated-glycoisoform of integrin alpha-3 (ITGA3) from the urine of bladder cancer patients was utilized for bladder cancer diagnosis. ITGA3 was recognized with its specific lectin, UEA-I, and Islam MK. et al. reported the detection of urinary ITGA3 glycoisoform with the use of UEA conjugated on nanoparticles (the ITGA3-UEA assay). The assay is capable of discriminating bladder cancer from clinically challenging age-matched benign conditions (*p* = 0.007) [67].

Thus, targeting fucosylation may be useful in both the treatment and diagnosis of bladder cancer.

## 6. Fucosylation in Prostate Cancer

Prostate cancer is the second most frequently diagnosed cancer among men and was the cause of an estimated 385,000 deaths worldwide in 2018 [68]. Glycosylation is a common post-translational modification of secreted proteins that play a key role in many important biological processes in prostate cancer [69,70].

Core-type fucosylation is the major type of glycosylation in prostate cancer [71], and the levels of core-fucosylated glycans are significantly increased in serum samples derived from prostate cancer patients compared with patients with benign prostate hyperplasia [72,73]. The fucosylation of glycan structures is higher in the serum samples derived from patients with metastatic prostate cancer than in healthy males, showing area under the curve (AUC) values greater than 0.9 in several specific *N*-glycans [74]. Fucosylation of glycans plays an important role in prostate cancer development and progression. TRAMP mice deficient in α1-3 FUT activity exhibited a lower incidence of prostate cancer formation and a lower rate of tumor progression, as evidenced by significantly smaller prostate weights [75]. Fucosylated glycopeptides are overexpressed in aggressive prostate cancer cell lines compared with nonaggressive prostate cancer cells [76,77].

FUT8 is considered to be the only FUT involved in core-fucosylation based on the fact that FUT8 knockout mice showed no oligosaccharide structures with core fucose [78]. Analysis of fucosylation of serum haptoglobin showed that core-type fucosylation is more characteristic in prostate cancer than in gastrointestinal cancer [71]. High expression of FUT8 and haptoglobin was observed in prostate cancer cell lines, and serum levels of fucosylated haptoglobin were associated with high-grade prostate cancer [79]. FUT8 overexpression in LNCaP cells increased cell migration, whereas FUT8 knockdown in PC3 cells decreased cell motility [80]. FUT8 was overexpressed in androgen-resistant LAPC4 cells compared with androgen-dependent LAPC4 cells, suggesting the functional role of fucosylated enzymes associated with aggressive prostate cancer [81].

As described above, fucosylation is upregulated in malignancies as compared with that in benign disease. Thus, the detection of fucosylated proteins could be a biomarker of urological malignancies, especially in prostate cancer. Prostate-specific antigen (PSA) is a glycoprotein that is commonly used as a biomarker for prostate cancer diagnosis. However, a single PSA test is not sufficient to distinguish between prostate cancer and benign prostate hyperplasia (BPH). To develop novel biomarkers, there is an unmet clinical need to refine the PSA test. PSA has one N-glycosylation site (Asn-69) to which various types of oligosaccharides can be attached [69,82]. In 2004, Ohyama et al. demonstrated that free serum PSA from patients with prostate cancer exhibits increased binding to Maackia amurensis agglutinin (MAA) lectin, which recognizes α2,3-linked sialic acid, compared with that in patients with BPH [83]. Since then, a number of reports have been published on PSA glycosylation. Fucosylation in PSA has been studied by several groups. Fukushima et al. demonstrated that there is an elevated expression of α1,2-Fucosylation of PSA during prostate carcinogenesis, using Trichosanthes japonica agglutinin-II (TJA-II) column chromatography [84]. Dwek et al. demonstrated that serum α1,2-Fucosylated PSA was significantly higher in men with prostate cancer than in men with BPH using an enzyme-linked immunosorbent lectin assay with Ulex europaeus (UEA-1) [85]. Li et al. demonstrated that the fucosylated total PSA was significantly increased in high-risk prostate cancer and correlated with the tumor Gleason score, using a magnetic bead-based immunoassay in which fucosylated total PSA was detected by Aleuria aurantia lectin (AAL) [86]. AAL exhibits broader specificity towards fucosylation, whereas PhoSL and Lens culinaris agglutinin (LCA) show specificity toward core-type fucosylated *N*-glycans [87]. Since a lectin generally has a weak binding affinity to fucose, the development of lectin-antibody ELISA for serum fucosylated markers is difficult due to the existence of abundant proteins in the sera. Ishikawa et al. developed microfluidic electrophoresis immunoassay systems to measure the α2,3-linked sialyl N-glycan-carrying PSA (S2,3PSA) using MAL lectin [88]. They found that the ratio of serum S2,3PSA to non-sialylated PSA could predict prostate cancer better than serum PSA. Fujita et al. showed that the ratio of serum fucosylated PSA could differentiate high-risk prostate cancer from biopsy-negative or high-risk prostate cancer using PhoSL to detect core-type fucosylated free PSA using a microcapillary electrophoresis-based immunoassay system [89]. When S2,3PSA and core-type fucosylated PSA (FucPSA) were simultaneously measured by automated micro-total immunoassay systems, S2,3PSA was not correlated with FucPSA in patients with prostate cancer. Thus, the simultaneous measurement of S2,3PSA and FucPSA (SF index) increased the accuracy of the detection of high-risk prostate cancer. The SF index showed good discrimination for high Gleason score cancer (AUC 0.842; 95%CI, 0.782–0.903), compared with the single PSA test (AUC 0.632, 95%CI 0.543–0.721), S2,3PSA (AUC 0.711, 95%CI 0.629–0.793), and FucPSA (AUC 0.738, 95%CI 0.657–0.819) [90]. Thus, fucosylated PSA, especially core-type fucosylated PSA, can be a useful biomarker for prostate cancer diagnosis.

## 7. Therapeutic Target of Fucosylation

Since fucosylation is associated with tumor growth and invasion, inhibition of fucosylation has been attempted to treat tumors. Fluorinated fucose analogs inhibit the activity of FUTs, including FUT8, and inhibit the proliferation of several types of cancer cells [91]. The L-fucose analog 2-fluoro-L-fucose (2FF) inhibits core fucosylation by interfering with the normal synthesis of GDP-fucose, resulting in the inhibition of cell proliferation and migration in liver cancer cell lines [92]. The development of specific FUT inhibitors or neutral antibodies for core fucose could lead to the development of a new class of drugs for cancer. Miyoshi et al. revealed that fucosylation controls the sensitivity to NK cells through regulating tumor-necrosis-factor-related apoptosis-inducing ligand (TRAIL) signaling-induced apoptosis. Although the increase in fucosylation plays important roles at an early stage of carcinogenesis, tumor cells could escape from NK-cell-mediated tumor surveillance by defucosylation induced by genetic mutation in several types of advanced cancers [93].

Soluble recombinant human TRAIL or agonistic antibodies targeting TRAIL receptors may be promising proapoptotic anti-tumor therapeutic agents in patients with several types of tumors, but many types of tumor cells have been shown to be resistant to TRAIL [94,95,96]. However, it is expected that combination therapy of TRAIL and demethylation agents could be promising immunotherapy. Further studies should clarify how fucosylation affects cancer immunology, and then TRAIL therapy could be a more effective means of treatment. Fucosylation is a promising target for cancer therapeutics.

## 8. Conclusions

In conclusion, fucosylation plays an important role in urological cancers. Fucosylated proteins could be a promising biomarker for urological malignancies, especially prostate cancer. Furthermore, elucidation of the biological function of fucosylation could result in the development of novel therapeutic targets. Further studies are needed in the near future in the field of fucosylation in urological malignancies.

## Figures and Tables

**Table 1:** Biological function of fucosyltransferases.

Fucosyltransferase	Type of Fucosylation	Function in Cancer	References
FUT1	α1-2 fucosylation	Migration, invasion, epithelial-mesenchymal transition, and drug resistance	[6,7,8]
FUT2	α1-2 fucosylation	Migration, invasion, and epithelial-mesenchymal transition (EMT)	[6]
FUT3	α1-3/α1-4 fucosylation	Activation of TGF-β signaling pathway	[9,10]
FUT4	α1-3/α1-4 fucosylation	Drug resistance, invasion, migration, EMT, and cell adhesion	[11,12,13]
FUT5	α1-3/α1-4 fucosylation	Activation of PI3K/Akt signaling pathway	[14]
FUT6	α1-3/α1-4 fucosylation	Activation of PI3K/Akt signaling pathway	[14]
FUT7	α1-3/α1-4 fucosylation	Proliferation, migration, invasion, and EMT; activation of MAPK and PI3K/Akt signaling pathway via EGFR	[15,16]
FUT8	α1-6 fucosylation	Invasion, migration, and EMT; activation of TGF-β and EGFR signaling pathway	[17,18,19,20,21,22]
FUT9	α1-3/α1-4 fucosylation	Cancer stemness	[23]
FUT10	No fucoysltransferase activity		
FUT11	No fucoysltransferase activity		

## Data Availability

Not applicable.
